# Necrological

**Published:** 1880-09

**Authors:** 


### NECROLOGICAL.

" Death is the crown of life:
Were death denied, poor man would live in vain."

We are pained to announce the death of Alfred Swaine Taylor, M. D., F. R. S. of London. Dr. Taylor's name is inseparably associated with the present state of Medical Jurisprudence. He was a graduate of St. Andrew's, and fellow of most of the learned and professional societies of Great Britain. He was a Professor at Guy's Hospital, and author of the great works that have immortalized his name.

